# A rapid review of current engagement strategies with people who use drugs in monitoring and reporting on substance use-related harms

**DOI:** 10.1186/s12954-023-00902-x

**Published:** 2023-11-14

**Authors:** Melissa Perri, Triti Khorasheh, David Edward-Ooi Poon, Nat Kaminski, Sean LeBlanc, Leticia Mizon, Ashley Smoke, Carol Strike, Pamela Leece

**Affiliations:** 1 Dalla Lana School of Public Health, University of Toronto, 480 University Ave, Suite 300, Toronto, ON M5G 1V2 Canada; 2 Health Promotion, Chronic Disease and Injury Prevention, Public Health Ontario, Toronto, ON Canada; 3 Ontario Network of People Who Use Drugs, ON, Canada; 4 MAP Centre for Urban Health Solutions, St. Michael’s Hospital, Toronto, ON Canada; 5 Department of Family and Community Medicine, University of Toronto, Toronto, ON Canada

**Keywords:** Data monitoring, Community engagement, Substance use-related harm, Data reporting, Opioids, Overdose crisis, Information systems, People who use drugs

## Abstract

**Background:**

The Canadian drug supply has significantly increased in toxicity over the past few years, resulting in the worsening of the overdose crisis. A key initiative implemented during this crisis has been data monitoring and reporting of substance use-related harms (SRH). This literature review aims to: (1) identify strategies used for the meaningful engagement of people who use drugs (PWUD) in local, provincial, and national SRH data system planning, reporting, and action and (2) describe data monitoring and reporting strategies and common indicators of SRH within those systems.

**Methods:**

We searched three academic and five gray literature databases for relevant literature published between 2012 and 2022. Team members who identify as PWUD and a librarian at Public Health Ontario developed search strings collaboratively. Two reviewers screened all search results and applied the eligibility criteria. We used Microsoft Excel for data management.

**Results:**

Twenty-two articles met our eligibility criteria (peer-reviewed *n* = 10 and gray literature reports *n* = 12); most used qualitative methods and focused on the Canadian context (*n* = 20). There were few examples of PWUD engaged as authors of reports on SRH monitoring. Among information systems involving PWUD, we found two main strategies: (1) community-based strategies (e.g., word of mouth, through drug sellers, and through satellite workers) and (2) public health-based data monitoring and communication strategies (e.g., communicating drug quality and alerts to PWUD). Substance use-related mortality, hospitalizations, and emergency department visits were the indicators most commonly used in systems of SRH reporting that engaged PWUD.

**Conclusion:**

This review demonstrates limited engagement of PWUD and silos of activity in existing SRH data monitoring and reporting strategies. Future work is needed to better engage PWUD in these processes in an equitable manner. Building SRH monitoring systems in partnership with PWUD may increase the potential impact of these systems to reduce harms in the community.

**Supplementary Information:**

The online version contains supplementary material available at 10.1186/s12954-023-00902-x.

## Background

The Canadian drug supply has significantly increased in toxicity over the past few years, driven by fentanyl and other synthetic opioids [1]. This increase contributes to a worsening crisis of fatal and non-fatal overdoses. Between January and December 2021, there were approximately 21 opioid-related deaths per day in Canada (annual rate of 19.3 per 100,000 population), including a 96% increase in the first year of the COVID-19 pandemic (April 2020–March 2021) compared to the year prior (April 2019–March 2020) [1]. Deaths involving stimulants have also remained high, representing 59% of accidental opioid-related deaths in 2021 [2]. Similar rates have been documented within the USA with over 80,000 overdose-related deaths occurring between 2019 and 2020 (annual rates of opioid-related deaths in 2020: 16.7 (Canada) and 21.4 (USA) per 100,000 population) [1, 3].

During this crisis, one of the key actions implemented across the USA, Canada, and other international jurisdictions has been the monitoring of data on substance use-related harms (SRH) (used herein to refer to morbidity and mortality from substance use, including but not limited to fatal and non-fatal overdose) to inform response efforts and strategies [4–7]. Robust data monitoring aims to create a better understanding of patterns and circumstances around SRH, which can be used to inform prevention and response strategies [4–7]. As such, national, provincial, and local data systems have been developed and implemented across Canada. The Canadian HIV AIDS Legal Network, the Canadian Association of People who use Drugs, and the Canadian Centre on Substance Use and Addictions, among others, have developed guidance for the meaningful engagement of people who use drugs (PWUD) in the design and delivery of any policy, program, and research that aims to address SRH [8, 9]. Despite this, there is little known about the uptake and use of engagement principles and practices with PWUD in SRH data system planning, reporting, and actions.

This rapid review aims to summarize the peer-reviewed and gray literature on practices used to engage PWUD in monitoring of SRH. Specifically, our objective was to identify strategies used to engage PWUD in local, provincial, and national SRH monitoring and reporting systems. As a secondary objective, we describe existing data monitoring and reporting strategies that include community engagement, as well as common indicators used within these systems. Understanding current practices and indicators will help improve the meaningful engagement of PWUD in local and provincial SRH data monitoring for action in Ontario, Canada. 

## Methods

We conducted a rapid review to document practices used to engage PWUD in local, provincial, and national systems to monitor SRH. Rapid literature review methods are often used to gain an understanding of what evidence exists about a particular topic and can be useful in developing research questions or methodologies [10]. Compared to a systematic review, rapid reviews may omit some steps (e.g., quality appraisal) for the purpose of timeliness. We carried out the search, selection, data extraction, and analyses between January 27 and February 24, 2022. Our team consisted of an interdisciplinary group of academics, physicians, harm reduction workers, and experts from the Ontario People Who Use Drugs Network (ONPUD). ONPUD members nominated academic partners with long-standing experience in community-based research with PWUD and ongoing meaningful relationships. Each step of the review process was developed and implemented with team members who identify as PWUD. Throughout this paper, we use the terms data monitoring and systems rather than surveillance, as among PWUD the term surveillance may also refer to police surveillance and is associated with experiences of harm [11, 12].

### Search strategy

The review consisted of both peer-reviewed and gray literature searches. A librarian at Public Health Ontario (PHO) provided support for developing search strings, which included concepts such as “people who use drugs,” “overdose,” “hospitalization,” and “surveillance” (see Additional file 1: Appendix 1 and Additional file 2: Appendix 2). We applied the search strategy to peer-reviewed databases including Medline, Embase, and PsycINFO. Using similar search strings, we searched the following gray literature: Ontario Public Health Units, International Public Health Resources, Canadian Health Departments and Agencies, US State Government Websites, and Google; we reviewed 20 pages of results per databases. We supplemented all database searches with hand searching of relevant resource reference pages and through recommendations of relevant documents from project stakeholders. The search strategy focused on North America, Australia, New Zealand, Europe, and other developed countries with a similar context around substance use-related harms.

### Eligibility criteria 

Eligibility criteria included: (1) English language texts; (2) peer-reviewed and gray literature research manuscripts or reports; (3) focused on people who use unregulated drugs; (4) described strategies to engage PWUD in data monitoring and reporting on SRH; and (5) were published between the years of 2012 and 2022 (current at the time of the search). The search included documents from the last ten years to ensure relevancy to current data monitoring and reporting processes. In Canada, OxyContin was delisted from provincial drug funding formularies as of March 2012. This coincides with the acceleration of fentanyl-related deaths from 25.8% of opioid-related deaths in Ontario in 2012 to nearly 90% by 2021 [13, 14]. Articles that did not include a description of strategies to engage PWUD in SRH data monitoring or indicator reporting were excluded. Commentaries, book chapters, and editorial articles were also ineligible.

### Study selection process

Two reviewers (MP, TK, DEOP, and/or PL) applied the eligibility criteria to the titles and abstracts in pairs, as outlined above. The team collaboratively reviewed 20 articles (10 peer-reviewed and 10 gray literature) to ensure consistency and to develop a common understanding of the eligibility criteria among all screeners. Articles that met the above eligibility criteria based on titles and abstracts subsequently underwent full-text screening. The review of full-text articles followed the same process as titles and abstracts. A third reviewer was involved in discussion of all discrepancies among screeners. We used a screening template in a Microsoft Excel Spreadsheet as a structure for data management with all articles, which included information such as the article title, abstract, and year, the search engine used to find the source, and reasons for exclusion.

### Data charting and synthesis

We used a structured table in Microsoft Excel to manage data extraction. One team member (MP or DEOP) reviewed each article and extracted the following information: location of work, data collection and analysis methods reported, and engagement strategies described in the text. We also extracted information pertaining to data monitoring and reporting strategies (e.g., what strategies were used, how PWUD were involved) and any indicators used for specific outcomes that were identified within the literature. The co-principal investigator (PL) reviewed each of the extracted articles to ensure consistency. Once the screening and extraction steps were complete for each article, the full team met three times to analyze and interpret the findings. We developed a typology to apply to the engagement strategies described in the included manuscripts that distinguishes the roles of PWUD, with definitions described in Table 1. Data analysis was guided by the outlined research questions and included grouping findings thematically. We reviewed each article was reviewed and relevant information was summarized using a thematic approach. This included grouping information based on similar themes/content which related to the objectives of the review.

**Table 1:** Typology of roles for engagement of people who use drugs in monitoring and reporting on substance use-related harms

Role	Description
Participants	Literature that included PWUD as participants in research studies where they shared experiences and perceptions surrounding a specific topic
Advisory Members	Literature that included PWUD as advisors on projects or documents. In these situations, PWUD provided broad feedback on the development and/or dissemination of specific topics. Engagement in this situation was not ongoing
Consultants	Literature that included PWUD in an established role and ongoing partnership with the organization that led the development of the document. In these situations, PWUD provided ongoing feedback on the development and dissemination of the document
Authors	Literature that included PWUD as authors on documents, indicating full involvement in the development and interpretation of the report, in addition to responsibility and accountability for the published material [15]

## Results

**Fig. 1:**
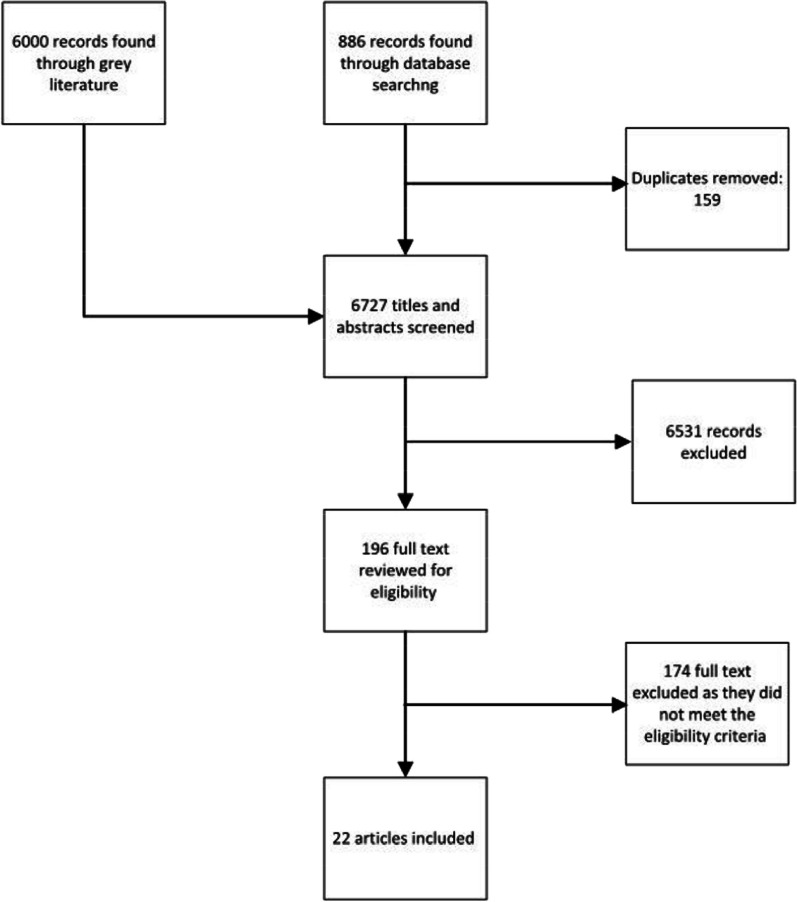
Prisma diagram of search and article inclusion process

Our search results consisted of 6727 records (886 from electronic databases, 6000 from gray literature, 159 duplicates removed), and 22 articles met the eligibility criteria. See Fig. 1 for PRISMA diagram. Ten of these articles were peer-reviewed manuscripts and 12 were gray literature reports (*n* = 12). The majority of literature focused on the Canadian context (*n* = 20) with one originating from the USA (*n* = 1) and another from North America (Canada, USA, Mexico) (*n* = 1). All articles were published between the years of 2015 and 2022, with most being published between 2019 and 2022.

There was limited discussion of specific strategies used to engage PWUD within SRH data monitoring and reporting systems. Levels of engagement among PWUD varied across the included articles. One article did not involve PWUD directly in the development of the review but included literature on information and risk communication strategies among PWUD on drug adulteration and quality, relevant SRH monitoring and reporting systems [16]. Table 2 provides an overview of each included article, its purpose, and the role of PWUD.

**Table 2:** Overview of included literature on data monitoring of substance use-related harms, purpose of the study/report, and engagement of people who use drugs

Article	Document type	Summary of purpose	Involvement of PWUD
Bardwell et al. [32]	Peer-Reviewed Paper	Examines the level of trust PWUD have in their drug dealers Provides perspectives on the potential for drug dealers to use DCTs for customers	Participants
Bardwell et al. [29]	Peer-Reviewed Paper	Examines the willingness to use various DCT among structurally-vulnerable PWUD	Participants
Betsos et al. [30]	Peer-Reviewed Paper	Explores drug sellers’ negotiation of and engagement with DCT	Participants
British Columbia Centre for Disease Control [16]	Report	A brief literature review conducted to examine the perceptions of people who use drugs on adulteration practices and drug quality assessment techniques	Participants
Buxton et al. [20]	Peer-Reviewed Paper	Describes the formation and surveillance system of the British Columbia Drug Overdose and Alert Partnership Uses examples of fentanyl-associated overdoses and deaths to describe the attributes that make the system effective	Consultants
Carroll et al. [9]	Peer-Reviewed Paper	Explores the social and relational factors that shape the current opioid overdose epidemic	Participants
Cook [31]	Report	Provides an overview of problematic substance use demonstrating the need for enhanced harm reduction services Presents options for addressing the current opioid crisis, including exploring the feasibility of supervised injection services	Consultants
Gomes et al. [26]	Report	Reviews the circumstances surrounding opioid-related deaths during the pandemic Reviews patterns of opioid-related mortality and morbidity to inform interventions	Authors
Gomes et al. [27]	Report	Describes the characteristics and healthcare patterns of people who died of an accidental opioid-related toxicity prior to and during the pandemic Focuses people who were experiencing homelessness in order to inform supportive approaches	Authors
Kolla and Penn [37]	Report	Provides evaluation of a program designed to reduce barriers to access of harm reduction equipment, supplies, and education, and to reduce social isolation Describes program linkages to health care and social services among PWUD, who were otherwise unconnected to services and care	Participants
Kolla and Strike [17]	Peer-Reviewed Manuscript	Examines the integration of people who sell drugs directly into harm reduction service provision, and their practices of care with other PWUD in their community	Participants and Advisory Members
Loyal and Buxton [33]	Report	Identifies methods and modes of communicating drug alerts, how they can be improved, and how they affect drug choice and use behaviours Explores how peers receive information about toxic drugs and the needs of those who do not access harm reduction supply distribution sites	Participants
Ontario Agency for Health Protection and Promotion et al. [18]	Report	Summarizes the characteristics of persons and the circumstances surrounding their deaths from completed investigations of confirmed opioid-related deaths	Advisory Members
Palamar et al. [34]	Peer-Reviewed Manuscript	Investigates the research gap between formal drug checking services and personal test kits Provides important information regarding the provision of these harm reduction initiatives and identifying potential areas for improvement	Participants
Parkinson et al. [19]	Report	Provides information related to substance use trends, drug-related health issues and risk behaviors/needs of PWUD Discusses barriers and supports to accessing health care and supportive services to improve the health of PWUD	Participants and Advisory Members
Region of Peel [22]	Report	Reports on the local response efforts focused on better understanding and monitoring and responding to local opioid use and overdose Discusses bringing together stakeholders, enforcement, and justice related to opioid/substance use around one table	Consultants
Scarfone et al. [28]	Peer-Reviewed Manuscript	Presents trends of samples analyzed early during DCS implementation, along with reported negative effects Identifies the prevalence of high-potency opioids in the unregulated drug supply as well as combinations with stimulants, benzodiazepines, and synthetic cannabinoids	Authors
Shepherd and Caldwell [23]	Report	Describes a comprehensive set of actions to prevent and respond to overdoses, focusing on actions at the local level Discussion combining the knowledge and expertise of PWUD, their family and friends, and people working in the field, with best practices and research	Consultants
Soukup-Baljak et al. [35]	Peer-Reviewed Manuscript	Describes the perspective of PWUD to characterize the practices used to assess the quality of street drugs and to reduce harms from adulterants Develops recommendations on how to effectively communicate drug alerts to PWUD	Participants
Wallace et al. [36]	Peer-Reviewed Paper	Examines the potential impacts of community DCS through a socio-ecological model, from the perspective of service users Evaluates harm reduction and social justice through effectiveness of DCS within the context of illicit drug criminalization, stigmatization, and the overdose crisis	Participants
Windsor-Essex Country Health Unit [24]	Report	Provides recommendations categorized under the Four Pillars approach Discusses the role for enforcement agencies and first responders to build partnerships for a safer and healthier community	Consultants
York Region Public Health [25]	Report	Describes strategies for harm reduction under the Four Pillars approach, with feedback from PWUD	Consultants

*PWUD* people who use drugs, *DCT* drug checking technologies, *DCS* drug checking services

### Role of people who use drugs within the literature

There were four main ways in which PWUD were engaged in SRH data monitoring and reporting systems: as participants in research studies, as advisory members, as consultants, or as co-authors. People may have been engaged in more than one role. Most of the academic literature included in this report engaged PWUD as participants (*n* = 11) in studies related to monitoring and reporting systems. Three articles engaged with PWUD as advisory members of the systems [17–19]. These articles generally noted that PWUD provided feedback on the content of the report. Six articles consulted PWUD [20–25]. This was clearly outlined throughout each article with indication on partnerships made with PWUD and key recommendations/content created specifically from the consulting groups. Lastly, three articles included PWUD as co-authors on the articles, such as the Harm Reduction Coordinating Committee (partly composed of community members with lived/living experiences), Ontario Drug Policy Research Network (ODPRN) (which involved a citizens’ panel and a Lived Experience Advisory Group), and the Toronto’s Drug Checking Service Working Group [18, 26–28].

### Data monitoring and reporting systems with engagement

The available information allows for the description of existing systems for monitoring and reporting that engaged PWUD. This included information pertaining to how community and public health stakeholders are facilitating SRH data monitoring and reporting and what indicators are being used across these systems. Peer-reviewed articles explored topics relating to drug sample analyses (e.g., drug checking services) and communication methods for sharing information among PWUD, including current indicators used in data monitoring systems. Among the gray literature reports, four reports described jurisdictional strategies and action plans aimed at reducing opioid or broader SRH (Toronto, Windsor-Essex, York Region, and Peel Region). These included community recommendations on timely and effective communication of drug alerts and online dissemination of information.

#### Community and network strategies

Eleven articles focused on community-based strategies for monitoring and reporting SRH [16, 29–37]. Ten articles mentioned monitoring and reporting of SRH through drug sellers (commonly referred to as “drug dealing” or “drug dealers”) [16, 17, 30, 31, 33–36]. Many articles (10 of 22, 45%) highlighted the role of drug sellers in both drug checking (i.e., testing drug samples) and in communicating drug quality, potency, and alerts to members of the community [16, 17, 29–36]. In qualitative studies, PWUD expressed key aspects of engaging with drug sellers for drug quality monitoring such as if the supply was gathered from a new source, if the color, potency, or texture was different, and whether they had heard of any overdose-related events in the community from the same batch of drugs [16, 31, 33, 34]. In an ethnographic study led by Betsos et al. (2021) in Vancouver, Canada, drug sellers explained that drug checking technologies helped support drug monitoring and communication to clients as it facilitated accessibility and reliability [30]. However, some drug sellers did not engage in drug checking technologies often because of the time commitment, limited availability of services, and limited confidence about drug composition.

Peer-based social networks were also described as a drug monitoring and communication community-based strategy. Soukup-Baljak et al. (2015) discussed the methods of communicating drug quality to PWUD in British Columbia, Canada, and found that all participants (*n* = 32) agreed that “word-of-mouth” strategies were most commonly used for gathering and providing information about drug quality [35]. In addition, the use of social networks and word of mouth was described as a key method of alerting peers about a toxic drug supply. In addition to highlighting the role of peers in monitoring and communication of SRH, people in this study also provided key recommendations to improve drug alert communication with the substance use community. Key recommendations from participants in this study included: use brief, simple language; improve timeliness of alerts; communicate using posters and social media; and include the number of overdoses involved.

Satellite workers represented a third community-driven strategy for the monitoring and communication of SRH. Satellite workers provide harm reduction specific services such as supplying people with harm reduction equipment and education within residential and shelter settings [17, 37]. Evaluations of satellite programs and workers in Toronto, Canada, highlight their effectiveness in facilitating information exchange among clients and broader community members. This research demonstrated that satellite workers have “mobile community intel regarding drug trends, quality, concentration, and reactions within the local drug markets, and issuing drug warnings” [37, p. 6]. Satellite workers gather information from the community they work with or from their own lived/living experiences and disseminate it accordingly (e.g., among community members or between programs and the community members served).

#### Public health strategies

Eight articles included in this review leveraged public health-related monitoring strategies for SRH [19–25, 33]. Half of these articles (*n* = 4) focused on local strategies and plans targeted at minimizing SRH (e.g., Peel Opioid Strategy: A Local Response) [22–25]. The articles focused on service-level strategies pertaining to communicating drug quality and alerts to PWUD, developing effective and equitable data monitoring systems for SRH, and highlighting the importance of cross-sectoral and interdisciplinary stakeholder involvement in the development and implementation of SRH-related interventions. Emphasis was placed on the development of local SRH data and communication systems.

The York Region Opioid Action Plan, for example, indicates that a local warning system aimed to share information about overdoses and other SRH should be developed and shared among varying health and social service providers [25]. This action plan also calls for the development of a risk assessment tool in association with the collection of both quantitative and qualitative data associated with opioid use and its related impacts in the region. PWUD acted as consultants in the development of this action plan and recommended the use of an online tool to disseminate information on drugs in the area. PWUD noted that the tool should:

- Provide anonymity or confidentiality.
- Be easy to use.
- Be a place to find archived and factual information.
- Be able to reach a large number of people.
- Be a place to report drug-related concerns (e.g., toxicity of drugs, defective harm reduction supplies).
- Be a place to share personal stories.

The recommendations from York Region also addressed the importance of incorporating indicators within monitoring of SRH that are informed by people with lived/living experiences (e.g., qualitative reports from the community, “people who know what’s going on”). Consultants noted that indicators such as emergency overdose visits or hospitalizations neglect to consider the realities of opioid-related harms in communities (e.g., opioid withdrawal and other substance use issues) and ignore the fact that many PWUD avoid hospital settings due to experiences of stigma and harm.

Similar strategies were discussed within the Toronto Overdose Action Plan: Prevention and Response, which also consulted people with lived/living experience [23]. Regarding monitoring, people with lived/living experience highlighted the importance of anonymity, using clear and neutral language (e.g., surveillance is associated with harm), and ensuring SRH-related information is disseminated quickly and in an accessible way (e.g., through using websites) [23]. The importance of disseminating SRH-related information in an accessible and equitable way was also discussed by Loyal et al. 2021 who consulted with service users and service providers in British Columbia, Canada, surrounding the communication of drug alerts [33]. Using literature reviews, interviews, and focus groups, this study highlighted that:

- Timeliness is a key consideration for the effectiveness of communication such as drug alerts.
- Neutral and trigger free language should be included in communication.
- Most jurisdictions globally (e.g., United Kingdom, Netherlands) have incorporated web or app based strategies for monitoring and communication of SRH.
- Poster communication is valuable but not as accessible as “word-of-mouth” communication which reaches groups not connected to health or social services. This indicates the need for varying forms of communication pertaining to SRH.
- Incorporating people with lived/living experience in the creation and dissemination of SRH communication is essential.

The Peel Opioid Strategy: A Local Response document [22] and a report by the Region of Waterloo Public Health [21] both provide examples of existing monitoring systems in each region. Within the Region of Peel in Ontario, Canada, Peel Public Health has developed a surveillance method which aims to disseminate relevant information on SRH (e.g., overdoses) and naloxone distribution, which is gathered from Peel Paramedics and varying stakeholders in the community (e.g., harm reduction service providers) [22]. In comparison, the Region of Waterloo in Ontario, Canada, has implemented the Overdose Monitoring, Alert, and Response System (OMARS), which refers to a committee comprised of various stakeholders that aims to provide relevant SRH information (e.g., drug alerts) to community members, the general public, and health and social service providers [21]. A final example of a monitoring system that was discussed in the included literature was the British Columbia Drug Overdose and Alert Partnership (DOAP) [20]. The DOAP represents an intersectoral data monitoring system that includes stakeholders from varying jurisdictions (e.g., individuals with lived/living experience, emergency health services, public health agencies, and research centers). Using a variety of data sources (e.g., Vancouver Coastal Health, BC Coroners Services), DOAP disseminates SRH information using a password protected website and responds to relevant concerns from varying stakeholders [20].

### Indicators and trends

Among systems that engaged PWUD, six articles included in this review investigated SRH trends in by region, composition of substances, or characterized the circumstances surrounding overdose events. Literature published over the last two years (2020 and 2021) focused on how trends were influenced by the COVID-19 pandemic [26, 27]. Three articles focused on the province of Ontario [18, 26, 27], and two focused on the cities of Waterloo [19, 21] and one focused on Toronto [28]. In these monitoring systems or reports with engagement, the most commonly used indicators in SRH reporting included substance use-related mortality (e.g., opioid-related deaths), hospitalizations (e.g., acute hospital admissions, mental health-related hospital admissions), and emergency department visits. Often, indicators were presented with various sub-analyses conducted surrounding unique characteristics of circumstances such as the age, gender, employment status, substances used (e.g., involvement of stimulants, benzodiazepines, alcohol), or housing situation of individuals who experienced substance-related mortality and manners of death among those who engaged in substance use.

In addition to the above documented SRH indicators, the relevant literature also focused on reporting the quality and consistency of substances in particular jurisdictions. Scarfone et al. (2022), for example, investigated the chemical composition of high-potency opioids, such as fentanyl, being consumed in Toronto, finding 87% also contained stimulants, while 21% of samples included benzodiazepine-type drugs, and 1% included synthetic cannabinoids [28]. At the provincial level in Ontario, Gomes et al. reported on the likelihood of non-prescription benzodiazepines including etizolam, being involved in opioid-related deaths (e.g., found in one in 20 opioid-related deaths prior to the pandemic increasing to more than one in four during the pandemic). Within the Region of Waterloo, Ontario, a survey conducted with 388 PWUD in 2017 also asked about patterns of substance use including use of opioids and crack [19].

## Discussion

This literature review found a total of 22 articles, ranging from peer-reviewed to gray literature reports that engaged PWUD in local, provincial/state, and national SRH data system reporting. We found little detail on the engagement strategies used, with several articles involving PWUD as research participants and few articles with involvement as authors.

Our results demonstrate that the limited engagement of PWUD as leaders in the development and implementation of SRH reports represents a major limitation in the existing literature and current documented practices. As an interdisciplinary group grounded in the principal of “nothing about us without us,” we believe that without meaningful involvement of PWUD in leadership positions (at all levels), existing SRH monitoring and reporting strategies will be unsuccessful in preventing morbidity and mortality, and risk furthering individual and system-level stigma and harm [38]. In contrast, many studies also describe how involving PWUD and peer workers (i.e., those with lived experience) in supervised consumption service operation supports implementation and sustainability [39–41]. Specifically, our team discussed that the failure to engage PWUD in the development and implementation of SRH data monitoring and communication systems leads to: (1) monitoring systems that are ineffective at capturing and disseminating information on substance-related morbidity and mortality in a timely manner; (2) the development of indicators that are not meaningful for people to act on reducing harms; and (3) monitoring and reporting systems that further oppress, stigmatize, and create added strain on PWUD and local community-run practices. For example, drug alert reports that are not disseminated in a timely manner create additional burden on communities to ensure updated information is provided to those who need it. Similarly, the lack of involvement of PWUD in larger data monitoring systems leaves community members unsure of where data is stored, captured, or how to access it.

Information on community-based/peer-led initiatives may not be publically available and thus not captured in our review, although we are aware of examples that address the issues outlined above. One example that community experts referred to was *The Nameless*, a community-based, peer-led harm reduction organization in St. Thomas, Ontario, that aims to provide support to help lessen harms associated with varying forms of poverty [42]. They work with a wide variety of people and engage in direct SRH monitoring and dissemination through communicating drug alerts to members of their organization and to other PWUD. Their data monitoring and dissemination strategy is run by peer networks and includes posting drug alerts using flyers, communicating them by word of mouth, via text, phone calls, and through social media platforms such as Facebook. This community-run data monitoring initiative has been a pillar in this rural community for sharing SRH trends, helping to minimize substance use-related harms across the jurisdiction.

Other initiatives such as the Real-Time Drug Alert and Response project [43], located in British Columbia, allow people to submit text reports of drug quality or overdose instances in the community and represents an innovative method that addresses some practice-based recommendations surrounding data monitoring. Systems such as these can help alleviate issues surrounding delays in drug alert communications, the lack of community-driven knowledge on drug quality and overdose events, and can help members of the substance use community connect with each other. Similar initiatives that are led by members of a specific community include Bad Date Reporting [44–46], used among people engaged in sex work to confidentially report any negative experiences to a larger system to help prevent further harm. However, evaluations of bad date reporting procedures in British Columbia, Canada, show that often, formal bad date reporting documents were inaccessible and that most people gained information about bad dates through peer networks [47].

There remains a need to further reduce barriers to engagement for PWUD in adapting and developing future SRH data monitoring systems. Our community-based team members have identified that to build the capacity of existing community networks larger government run monitoring systems must: (1) increase funding to community-led groups to advance strategies currently being used for SRH data monitoring and communication; (2) remove barriers faced by PWUD such as stigmatized hiring practices (e.g., criminal record checks); (3) employ and provide training to PWUD as core stakeholders in provincial, territorial, and federal SRH data monitoring and communication agencies; (4) hold organizations accountable to existing best practice guidelines for engaging PWUD; and (5) commit to working across relevant stakeholders to use data for action to reduce harm associated with substance use. These suggestions reflect the application of Canadian best practice guidance on meaningful engagement of PWUD, addressing issues such as funding, criminalization, and employment, “to ensure equitable and just opportunities within program and policy domains that affect their lives” [48].

Existing guidelines and research addresses strategies that can help facilitate the engagement of people with lived/living experience in the substance use sector [8]. For example, Greer and colleagues worked with PWUD in British Columbia, Canada, to provide an overview of factors that assist in their engagement in substance use-related policy and practices [49]. Key methods of ensuring effective and equitable engagement was through, payment of PWUD for their expertise, clear definition of roles for PWUD and involvement of peer-based organizations in substance use-related programming and policy development. These findings are echoed by Brown et al. (2019) who reported findings from a 5-year collaborative study with peer-led organizations aimed at identifying barriers and enablers for meaningful engagement in substance use-related research [50]. Key enablers for meaningful engagement in research included flexibility in the development of research projects, continual demonstration of support and commitment from research partners to community members, and actively working toward mobilizing expert knowledge into research findings that influence substance use practice and policy.

PWUD face multifaceted forms of oppression by existing systems that drive current SRH monitoring and communication. In addition, people with living/lived experience leading work within local systems are under immense pressure and face high levels of trauma and grief from the continued loss of friends, family members, and peers [50, 51]. To advance the engagement of PWUD in SRH monitoring and minimize harm perpetuated from existing systems, community-run monitoring and communication systems must be given support to increase their capacity. This paper is the first step of this team’s larger goal in co-designing a framework and toolkit of resources to guide meaningful engagement of PWUD in local and provincial SRH surveillance in Ontario.

Further research is needed on how best to operationalize expert knowledge in a way that is equitable and truly meaningful in monitoring and reporting of SRH. Effort must also be placed on shifting existing ideologies surrounding SRH data to allow PWUD to inform, develop, disseminate, and take ownership of monitoring and dissemination systems. As an interdisciplinary group of experts, we note the following considerations for future work in this area based on existing evidence and practice: (1) increase networking and support among relevant SRH data monitoring stakeholders (e.g., coalitions of PWUD and coroners’ offices); (2) create processes for collecting and disseminating SRH that can be used by all community and public health-based organizations; and (3) develop an interactive map which shows drug alerts in different jurisdictions that can be accessed provincially by coalitions of PWUD (e.g., ODMAP.org used by public health and safety in the USA and the Toxic Drug and Public Health Alerts in British Columbia) [52, 53]. For collection or use of data with Indigenous communities, we acknowledge that specific, Indigenous-led partnerships will be essential, as well as processes for community data ownership and control, guided by community ways of knowing and supporting health around substance use.

### Limitations

Even though this literature review was carried out using rigorous methods, it has limitations. Primarily, a large portion of literature included in this review was based in Canada within the provinces of Ontario and British Columbia. Including a search of local public health unit websites in Ontario likely over-represented information in this context (6 of 22 records, 27%); however, all other searching encompassed provincial/state, national and international levels. This focus in Ontario and British Columbia does not speak to current strategies which may be used across North America or in other parts of Canada, for the engagement of PWUD in data monitoring and reporting. Similarly, literature included here was limited to the English language and to publically available information (e.g., some community-based organizations may not have this information publically available). The search was completed at an earlier stage of a larger project and the capacity to update the search was limited. Further, public health resources at the national, province/state, and local level were largely consumed with response to the COVID-19 between 2020 and early 2023. While there have been resources for interventions to mitigate SRH during the pandemic, there has not been a focus on evolving data monitoring systems during this period.

In addition, there are a variety of different strategies which were highlighted in this review but our search did not identify information on the impact or effectiveness of systems for SRH data monitoring. Finally, although out of scope of this review, there were no methodological assessments completed of included studies. Despite this, our review presents unique findings surrounding current engagement of PWUD in SRH data monitoring with leadership from team members with lived/living experience of substance use.

## Conclusions

Effectively engaging PWUD in SRH data monitoring and reporting is essential to improve effectiveness and mitigate ongoing harms associated with the current overdose crisis. There remains limited engagement of PWUD as authors or leaders in existing data monitoring and reporting practices. Currently, data monitoring and reporting strategies which take place in the community and within broader public health sectors are siloed. Considerations for how to effectively engage PWUD in these processes are needed and to improve capacity of existing community-run data monitoring and communication systems.

### Supplementary Information

**Additional file 1**. Appendix 1.

**Additional file 2**. Appendix 2.

## Data Availability

Not applicable.
