# Supplementary material for: A rapid review of current engagement strategies with people who use drugs in monitoring and reporting on substance use-related harms
Source: Harm Reduct J. 2023 Nov 14;20:169. doi: 10.1186/s12954-023-00902-x (PMC10648706; doi:10.1186/s12954-023-00902-x)
Supplement: Supplementary file 1 — Additional file 1. Appendix 1. [file 12954_2023_902_MOESM1_ESM.docx]

Appendix 1: Search Strings for Peer-review Databases

MEDLINE
Ovid MEDLINEI ALL 1946 to February 23, 2022

| # | Searches | Results |
| --- | --- | --- |
| 1 | *Drug Overdose/ or Heroin Dependence/ or Morphine Dependence/ or Opium Dependence/ or Amphetamine-Related Disorders/ or Cocaine Smoking/ or Cocaine-Related Disorders/ or Drug Misuse/ or Drug Users/ or Inhalant Abuse/ or Narcotic-Related Disorders/ or *Opiate Overdose/ or Prescription Drug Misuse/ or Prescription Drug Overuse/ or Substance Abuse, Intravenous/ or Substance Abuse, Oral/ or Substance-Related Disorders/ | 144657 |
| 2 | Amphetamines/ae, po, to or Codeine/ae, po, to or Dihydrophine/ or Ethylmorphine/ae, po, to or exp Analgesics, Opioid/ae, po, to or Fentanyl/ae, po, to or Heroin/ae, po, to or Hydrocodone/ae, po, to or Hydromorphone/ae, po, to or Morphine Derivatives/ae, po, to or Morphine/ae, po, to or Oxycodone/ae, po, to or Oxymorphone/ae, po, to or Thebaine/ae, po, to or N-Methyl-3,4-methylenedioxyamphetamine/ae, po, to or Cocaine/ae, po, to or Crack Cocaine/ae, po, to or Designer Drugs/ae, po, to or Narcotics/ae, po, to | 34555 |
| 3 | ((((club or designer or illicit or illegal or intravenous or inject* or party or recreational or street) adj2 drug?) or amphetamine* or benzodiazepine* or narcotics or MDMA or molly or ecstasy or heroin or speed or codeine or copiate* or ((new or novel) adj1 "psychoactive substance*") or opioid* or polydrug* or polypharma* or polysubstance* or acetylfentan#l or avinza or carfentan#l or codeine or diacetylmorph#ne or diamorphine or dihydromorph#ne or ethylmorph#ne or fentan#l* or heroin or hydrocod#ne or hydromorph#ne or kadian or morphine or opana or opiate* or opioid* or opium or oxycod#ne or oxycontin or oxymorph#ne or percocet or phentan#l or thebaine or vicodin or "W-18") adj8 ("use" or user* or addict* or misuse or abuse)).ti,kw,kf. or (((((club or designer or illicit or illegal or intravenous or inject* or party or recreational or street) adj2 drug?) or amphetamine* or benzodiazepine* or narcotics or MDMA or molly or ecstasy or heroin or speed or codeine or copiate* or ((new or novel) adj1 "psychoactive substance*") or opioid* or polydrug* or polypharma* or polysubstance* or acetylfentan#l or avinza or carfentan#l or codeine or diacetylmorph#ne or diamorphine or dihydromorph#ne or ethylmorph#ne or fentan#l* or heroin or hydrocod#ne or hydromorph#ne or kadian or morphine or opana or opiate* or opioid* or opium or oxycod#ne or oxycontin or oxymorph#ne or percocet or phentan#l or thebaine or vicodin or "W-18") adj8 ("use" or user* or addict* or misuse or abuse)).ab. not medline.st.) | 37002 |
| 4 | Emergency Shelter/ or Group Homes/ or Halfway Houses/ or Homeless Persons/ or Homeless Youth/ or Housing Instability/ or Public Housing/ or Residential Facilities/ or ("no fixed address" or "sleeping rough" or "street involved" or "street living" or "the? street" or "the? streets" or ((facility or facilities or centre or centres or center or centers or program*) adj3 (residen* or "live in")) or ((temporar* or precarious* or provisional* or vulnerab* or unstab* or instab* or stable* or stability or insecur* or secur* or transient* or transitory) adj5 accomodation*) or ((street or displaced or transient*) adj2 (youth* or people* or person* or individual* or men or women or teen*)) or encampment* or homeless* or hostel or hostels or shelters or underhoused or unhoused).kf,kw,ti. | 28064 |
| 5 | *Codeine/ or exp *Analgesics, Opioid/ or *Fentanyl/ or *Heroin/ or *Hydrocodone/ or *Hydromorphone/ or *Oxycodone/ or *Oxymorphone/ or *Designer Drugs/ or *Drug Overdose/ or *Narcotics/ | 97656 |
| 6 | or/1-5 | 272837 |
| 7 | Community-Based Participatory Research/ or Citizen Science/ or Community Networks/ or Community Participation/ or Cooperative Behavior/ or Decision Making, Shared/ or Focus Groups/ or Patient Reported Outcome Measures/ or Peer Group/ or Stakeholder Participation/ or "Surveys and Questionnaires"/ or (((collaborative* or community* or participatory or stakeholder*) adj design) or ((community-based or community-driven) adj2 research*) or "nothing about us without us" or "Denver principle*" or GIPA or MIPA or ((client* or communit* or meaningful or patient* or public or stakeholder* or user*) and (engag* or incorporat* or integrat* or involv* or insight* or expertise))).ti,kw,kf. or ((client* or communit* or meaningful or patient* or public or stakeholder* or user*) adj3 (engag* or incorporat* or integrat* or involv* or insight* or expertise)).ab. | 800890 |
| 8 | Cause of Death/ or "International Classification of Diseases"/ or Epidemiological Monitoring/ or Medical Records Systems, Computerized/ or Public Health Informatics/ or Registries/ or Statistics as Topic/ or Adverse Drug Reaction Reporting Systems/ or Emergency Medical Services/ or Advanced Trauma Life Support Care/ or Emergency Medical Dispatch/ or Emergency Medical Service Communication Systems/ or Emergency Police Dispatcher/ or Emergency Service, Hospital/ or Trauma Centers/ or Emergency Services, Psychiatric/ or Poison Control Centers/ or "Transportation of Patients"/ or Ambulance Diversion/ or Ambulances/ or Air Ambulances/ or "International Classification of Diseases"/ or Population Surveillance/mt or Public Health Surveillance/mt or Registries/ or Sentinel Surveillance/mt or "Coroners and Medical Examiners"/sn, td, ut or Data Collection/mt or *Population Surveillance/ or *Public Health Surveillance/ or *Sentinel Surveillance/ or *Data Collection/ | 479108 |
| 9 | ((("911" or "999" or ambulance* or (emergency adj2 service*) or (poison adj2 (center* or centre*)) or prehospital) adj5 (call* or client or numbers or patient* or response* or statistics or "use" or usage or visit*)) or "emergency response system*" or (((emergency adj2 (department or room)) or "accident and emergency") adj5 (client or number* or patient* or response* or statistic* or "use" or usage or visit*)) or ((active or outbreak* or enhanced or population or "public health" or real-time or sentinel or syndrom*) adj2 surveillance)).kf,kw,ti,ab. or (((("911" or "999" or ambulance* or (emergency adj2 service*) or (poison adj2 (center* or centre*)) or prehospital) adj5 (call* or client or numbers or patient* or response* or statistics or "use" or usage or visit*)) or "emergency response system*" or (((emergency adj2 (department or room)) or "accident and emergency") adj5 (client or number* or patient* or response* or statistic* or "use" or usage or visit*)) or ((active or outbreak* or enhanced or population or "public health" or real-time or sentinel or syndrom*) adj2 surveillance)).ab. not medline.st.) | 81160 |
| 10 | 8 or 9 | 524413 |
| 11 | 6 and 7 and 10 | 832 |
| 12 | ((user* or client*) adj3 (involv* or incorporat* or target* or rapport* or engag* or consult or consulting or consultation)).ab,ti,kw. | 8464 |
| 13 | 6 and 10 and 12 | 37 |
| 14 | (((user* or client*) adj3 (involv* or incorporat* or target* or rapport* or engag* or consult or consulting or consultation)) and (counts or "reaching out" or surveillance or enumerating or track or tracking)).ab,ti,kw. | 393 |
| 15 | 6 and 14 | 18 |
| 16 | 11 or 13 or 15 | 861 |
| 17 | (exp Africa/ or exp Caribbean Region/ or exp Central America/ or exp Latin America/ or exp South America/ or exp Asia/ or Mexico/ or Developing Countries/) not (North America/ or exp Canada/ or exp United States/ or exp Australia/ or New Zealand/ or exp Europe/ or exp Developed Countries/) | 1374981 |
| 18 | 16 not 17 | 788 |
| 19 | limit 18 to last 10 years | 424 |
| 20 | limit 19 to english | 414 |
| 21 | remove duplicates from 20 | 413 |

**Embase
Ovid Embase <1974 to 2022 February 23>**

| # | Searches | Results |
| --- | --- | --- |
| 1 | *drug overdose/ or *opiate overdose/ or drug dependence/ or amphetamine dependence/ or benzodiazepine dependence/ or cocaine dependence/ or drug misuse/ or methamphetamine dependence/ or multiple drug abuse/ or narcotic dependence/ or heroin dependence/ or morphine addiction/ or opiate addiction/ or "drug use"/ or "recreational drug use"/ or substance abuse/ or inhalant abuse/ or drug abuse/ or drug induced disease/ or medication overuse/ or prescription drug misuse/ | 364526 |
| 2 | narcotic agent/ae, to or buprenorphine/ae, to or codeine/ae, to or diamorphine/ae, to or dihydromorphine/ae, to or hydrocodone/ae, to or hydromorphone/ae, to or oxycodone/ae, to or opiate/ae, to or cocaine/ae, to or morphine/ae, to or fentanyl derivative/ae, to or carfentanil/ae, to or fentanyl/ae, to or amphetamine/ae, to or morphine derivative/ae, to or oxymorphone/ae, to or thebaine/ae, to or midomafetamine/ae, to or designer drug/ae, to or illicit drug/ae, to or recreational drug/ae, to or street drug/ae, to or ethylmorphine/ae, to | 41463 |
| 3 | ((((club or designer or illicit or illegal or intravenous or inject* or party or recreational or street) adj2 drug?) or amphetamine* or benzodiazepine* or narcotics or MDMA or molly or ecstasy or heroin or speed or codeine or copiate* or ((new or novel) adj1 "psychoactive substance*") or opioid* or polydrug* or polypharma* or polysubstance* or acetylfentan#l or avinza or carfentan#l or codeine or diacetylmorph#ne or diamorphine or dihydromorph#ne or ethylmorph#ne or fentan#l* or heroin or hydrocod#ne or hydromorph#ne or kadian or morphine or opana or opiate* or opioid* or opium or oxycod#ne or oxycontin or oxymorph#ne or percocet or phentan#l or thebaine or vicodin or "W-18") adj8 ("use" or user* or addict* or misuse or abuse)).ti,kw,kf. or (((((club or designer or illicit or illegal or intravenous or inject* or party or recreational or street) adj2 drug?) or amphetamine* or benzodiazepine* or narcotics or MDMA or molly or ecstasy or heroin or speed or codeine or copiate* or ((new or novel) adj1 "psychoactive substance*") or opioid* or polydrug* or polypharma* or polysubstance* or acetylfentan#l or avinza or carfentan#l or codeine or diacetylmorph#ne or diamorphine or dihydromorph#ne or ethylmorph#ne or fentan#l* or heroin or hydrocod#ne or hydromorph#ne or kadian or morphine or opana or opiate* or opioid* or opium or oxycod#ne or oxycontin or oxymorph#ne or percocet or phentan#l or thebaine or vicodin or "W-18") adj8 ("use" or user* or addict* or misuse or abuse)).ab. not embase.st.) | 75990 |
| 4 | *codeine/ or *diamorphine/ or *fentanyl/ or *hydrocone/ or *hydromophone/ or *oxycodone/ or *oxymorphone/ or *narcotic agent/ or *designer drug/ or *illicit drug/ or *recreational drug/ or *street drug/ | 46966 |
| 5 | emergency shelter/ or halfway person/ or exp homeless person/ or residential home/ or ("no fixed address" or "sleeping rough" or "street involved" or "street living" or "the? street" or "the? streets" or ((facility or facilities or centre or centres or center or centers or program*) adj3 (residen* or "live in")) or ((temporar* or precarious* or provisional* or vulnerab* or unstab* or instab* or stable* or stability or insecur* or secur* or transient* or transitory) adj5 accomodation*) or ((street or displaced or transient*) adj2 (youth* or people* or person* or individual* or men or women or teen*)) or encampment* or homeless* or hostel or hostels or shelters or underhoused or unhoused).kf,kw,ti. | 26887 |
| 6 | or/1-5 | 479333 |
| 7 | citizen science/ or community care/ or community participation/ or cooperation/ or participatory research/ or shared decision making/ or patient-reported outcome/ or peer group/ or stakeholder engagement/ or questionnaire/ or (((collaborative* or community* or participatory or stakeholder*) adj design) or ((community-based or community-driven) adj2 research*) or "nothing about us without us" or "Denver principle*" or GIPA or MIPA or ((client* or communit* or meaningful or patient* or public or stakeholder* or user*) and (engag* or incorporat* or integrat* or involv* or insight* or expertise))).ti,kw,kf. or ((client* or communit* or meaningful or patient* or public or stakeholder* or user*) adj3 (engag* or incorporat* or integrat* or involv* or insight* or expertise)).ab. | 1180175 |
| 8 | ((("911" or "999" or ambulance* or (emergency adj2 service*) or (poison adj2 (center* or centre*)) or prehospital) adj5 (call* or client or numbers or patient* or response* or statistics or "use" or usage or visit*)) or "emergency response system*" or (((emergency adj2 (department or room)) or "accident and emergency") adj5 (client or number* or patient* or response* or statistic* or "use" or usage or visit*)) or ((active or outbreak* or enhanced or population or "public health" or real-time or sentinel or syndrom*) adj2 surveillance)).kf,kw,ti,ab. or (((("911" or "999" or ambulance* or (emergency adj2 service*) or (poison adj2 (center* or centre*)) or prehospital) adj5 (call* or client or numbers or patient* or response* or statistics or "use" or usage or visit*)) or "emergency response system*" or (((emergency adj2 (department or room)) or "accident and emergency") adj5 (client or number* or patient* or response* or statistic* or "use" or usage or visit*)) or ((active or outbreak* or enhanced or population or "public health" or real-time or sentinel or syndrom*) adj2 surveillance)).ab. not embase.st.) | 124445 |
| 9 | epidemiological surveillance/ or active surveillance/ or passive surveillance/ or population surveillance/ or public health surveillance/ or sentinel surveillance/ or monitoring/ or online monitoring/ or health care surveillance/ or patient monitoring/ or social media monitoring/ or register/ or patient registry/ or death registry/ or disease registry/ or "cause of death registry"/ or "international classification of diseases"/ or medical informatics/ or emergency health service/ or emergency medical dispatch/ or hospital emergency service/ or psychiatric emergency service/ or medical record/ or electronic medical record/ or statistics/ or pharmacovigilance/ or poison center/ or ambulance diversion/ or ambulance transportation/ or drug surveillance program/ or coroner/ or patient transport/ or emergency care/ or advanced trauma life support/ | 1166717 |
| 10 | 8 or 9 | 1251360 |
| 11 | 6 and 7 and 8 | 578 |
| 12 | ((user* or client*) adj3 (involv* or incorporat* or target* or rapport* or engag* or consult or consulting or consultation)).ab,ti,kw. | 10349 |
| 13 | 6 and 10 and 12 | 71 |
| 14 | (((user* or client*) adj3 (involv* or incorporat* or target* or rapport* or engag* or consult or consulting or consultation)) and (counts or "reaching out" or surveillance or enumerating or track or tracking)).ab,ti,kw. | 494 |
| 15 | 6 and 14 | 17 |
| 16 | 11 or 13 or 15 | 651 |
| 17 | (exp Africa/ or exp Asia/ or exp "South and Central America"/ or exp Mexico/ or developing country/) not (North America/ or Canada/ or United States/ or exp "Australia and New Zealand"/ or exp Europe/ or developed country/) | 1699264 |
| 18 | 16 not 17 | 608 |
| 19 | limit 18 to conference abstracts | 202 |
| 20 | 18 not 19 | 406 |
| 21 | limit 20 to last 10 years | 267 |
| 22 | limit 21 to english language | 264 |
| 23 | remove duplicates from 22 | 259 |

**PsycINFO
APA PsycInfo <1806 to February Week 2 2022>**

| # | Searches | Results |
| --- | --- | --- |
| 1 | *drug overdose/ or drug abuse/ or "substance use disorder"/ or inhalant abuse/ or polydrug abuse/ or heroin addiction/ or drug usage/ or "opioid use disorder"/ or drug dependency/ or intravenous drug usage/ or heroin addiction/ or morphine dependence/ | 90207 |
| 2 | exp *cocaine/ or *designer drugs/ or *heroin/ or *narcotic drugs/ or exp *opiates/ | 35217 |
| 3 | ((((club or designer or illicit or illegal or intravenous or inject* or party or recreational or street) adj2 drug?) or amphetamine* or benzodiazepine* or narcotics or MDMA or molly or ecstasy or heroin or speed or codeine or copiate* or ((new or novel) adj1 "psychoactive substance*") or opioid* or polydrug* or polypharma* or polysubstance* or acetylfentan#l or avinza or carfentan#l or codeine or diacetylmorph#ne or diamorphine or dihydromorph#ne or ethylmorph#ne or fentan#l* or heroin or hydrocod#ne or hydromorph#ne or kadian or morphine or opana or opiate* or opioid* or opium or oxycod#ne or oxycontin or oxymorph#ne or percocet or phentan#l or thebaine or vicodin or "W-18") adj8 ("use" or user* or addict* or misuse or abuse)).ti,id. or (((((club or designer or illicit or illegal or intravenous or inject* or party or recreational or street) adj2 drug?) or amphetamine* or benzodiazepine* or narcotics or MDMA or molly or ecstasy or heroin or speed or codeine or copiate* or ((new or novel) adj1 "psychoactive substance*") or opioid* or polydrug* or polypharma* or polysubstance* or acetylfentan#l or avinza or carfentan#l or codeine or diacetylmorph#ne or diamorphine or dihydromorph#ne or ethylmorph#ne or fentan#l* or heroin or hydrocod#ne or hydromorph#ne or kadian or morphine or opana or opiate* or opioid* or opium or oxycod#ne or oxycontin or oxymorph#ne or percocet or phentan#l or thebaine or vicodin or "W-18") adj8 ("use" or user* or addict* or misuse or abuse)).ab. not psycinfo.st.) | 39495 |
| 4 | assisted living/ or dormitories/ or group homes/ or retirement communities/ or shelters/ or homeless/ or homeless mentally ill/ or residential care institutions/ or ("no fixed address" or "sleeping rough" or "street involved" or "street living" or "the? street" or "the? streets" or ((facility or facilities or centre or centres or center or centers or program*) adj3 (residen* or "live in")) or ((temporar* or precarious* or provisional* or vulnerab* or unstab* or instab* or stable* or stability or insecur* or secur* or transient* or transitory) adj5 accomodation*) or ((street or displaced or transient*) adj2 (youth* or people* or person* or individual* or men or women or teen*)) or encampment* or homeless* or hostel or hostels or shelters or underhoused or unhoused).ti,id. | 26711 |
| 5 | or/1-4 | 150945 |
| 6 | client participation/ or cooperation/ or community involvement/ or exp experimental methods/ or focus group/ or social networks/ or online social networks/ or peer relations/ or peers/ or patient reported outcome measures/ or surveys/ or consumer surveys/ or mail surveys/ or online surveys/ or telephone surveys/ or questionnaires/ or questionnaires/ or stakeholder/ | 151875 |
| 7 | (((collaborative* or community* or participatory or stakeholder*) adj design) or ((community-based or community-driven) adj2 research*) or "nothing about us without us" or "Denver principle*" or GIPA or MIPA or ((client* or communit* or meaningful or patient* or public or stakeholder* or user*) and (engag* or incorporat* or integrat* or involv* or insight* or expertise))).ti,id. or ((client* or communit* or meaningful or patient* or public or stakeholder* or user*) adj3 (engag* or incorporat* or integrat* or involv* or insight* or expertise)).ab. | 61906 |
| 8 | 6 or 7 | 206633 |
| 9 | ((("911" or "999" or ambulance* or (emergency adj2 service*) or (poison adj2 (center* or centre*)) or prehospital) adj5 (call* or client or numbers or patient* or response* or statistics or "use" or usage or visit*)) or "emergency response system*" or (((emergency adj2 (department or room)) or "accident and emergency") adj5 (client or number* or patient* or response* or statistic* or "use" or usage or visit*)) or ((active or outbreak* or enhanced or population or "public health" or real-time or sentinel or syndrom*) adj2 surveillance)).id,ti,ab. or (((("911" or "999" or ambulance* or (emergency adj2 service*) or (poison adj2 (center* or centre*)) or prehospital) adj5 (call* or client or numbers or patient* or response* or statistics or "use" or usage or visit*)) or "emergency response system*" or (((emergency adj2 (department or room)) or "accident and emergency") adj5 (client or number* or patient* or response* or statistic* or "use" or usage or visit*)) or ((active or outbreak* or enhanced or population or "public health" or real-time or sentinel or syndrom*) adj2 surveillance)).ab. not psycinfo.st.) | 7854 |
| 10 | "debriefing (psychological)"/ or "international classification of diseases"/ or *data collection/ or *disease collection/ or *drug overdoses/ or crisis intervention services/ or crisis intervention/ or electronic health records/ or emergency personnel/ or emergency services/ or facility admission/ or fire fighters/ or first responders/ or hospital admission/ or hospital discharge/ or hospitalization/ or hot line services/ or institutional release/ or medical records/ or paramedical sciences/ or paramedics/ or police personnel/ or psychiatric hospital admission/ or psychiatric hospitalization/ or rescue workers/ or suicide prevention centers/ or suicide prevention/ or treatment facilities/ or walk in clinics/ | 68862 |
| 11 | 9 or 10 | 73116 |
| 12 | 5 and 8 and 11 | 333 |
| 13 | ((user* or client*) adj3 (involv* or incorporat* or target* or rapport* or engag* or consult or consulting or consultation)).ab,ti,id. | 8875 |
| 14 | 5 and 13 and 11 | 45 |
| 15 | (((user* or client*) adj3 (involv* or incorporat* or target* or rapport* or engag* or consult or consulting or consultation)) and (counts or "reaching out" or surveillance or enumerating or track or tracking)).ab,ti,id. | 162 |
| 16 | 5 and 15 | 18 |
| 17 | 12 or 14 or 16 | 356 |
| 18 | limit 17 to last 10 years | 216 |
| 19 | limit 18 to english language | 208 |
| 20 | remove duplicates from 19 | 208 |
