# Supplementary material for: A rapid review of current engagement strategies with people who use drugs in monitoring and reporting on substance use-related harms
Source: Harm Reduct J. 2023 Nov 14;20:169. doi: 10.1186/s12954-023-00902-x (PMC10648706; doi:10.1186/s12954-023-00902-x)
Supplement: Supplementary file 2 — Additional file 2. Appendix 2. [file 12954_2023_902_MOESM2_ESM.docx]

Appendix 2: Search Strings for Grey Literature

hospitalization OR hospitalisation OR "emergency room" OR "emergency department" OR "accident and emergency" counts OR "reaching out" OR surveillance OR enumerating OR track OR tracking "rough sleeper" OR "sleeping rough" OR homeless OR homelessness OR unhoused

hospitalization OR hospitalisation OR "emergency room" OR "emergency department" OR "accident and emergency" counts OR "reaching out" OR surveillance OR enumerating OR track OR tracking "drug users" OR "people who use drugs" OR "drug addicts" OR "people who inject drugs" OR "people who use injection drugs" OR "substance users"

hospitalization OR hospitalisation OR "emergency room" OR "emergency department" OR "accident and emergency" counts OR "reaching out" OR surveillance OR enumerating OR track OR tracking "rooming house" OR shelters OR encampment OR "community housing" OR "public housing"

hospitalization OR hospitalisation OR "emergency room" OR "emergency department" OR "accident and emergency" counts OR "reaching out" OR surveillance OR enumerating OR track OR tracking "opioid users" OR "opiate users" OR "heroin users" OR "fentanyl users" OR "opioid addiction" OR "opiate addiction" OR "heroin addiction" OR "fentanyl addiction"

EMS OR paramedics OR "ambulance calls" OR "911 calls" OR "first responders" counts OR "reaching out" OR surveillance OR enumerating OR track OR tracking "rough sleeper" OR "sleeping rough" OR homeless OR homelessness OR unhoused

EMS OR paramedics OR "ambulance calls" OR "911 calls" OR "first responders" counts OR "reaching out" OR surveillance OR enumerating OR track OR tracking "drug users" OR "people who use drugs" OR "Drug addicts" OR "people who inject drugs" OR "people who use injection drugs" OR "substance users"

EMS OR paramedics OR "ambulance calls" OR "911 calls" OR "first responders" counts OR "reaching out" OR surveillance OR enumerating OR track OR tracking "rooming house" OR shelters OR encampment OR "community housing" OR "public housing"

EMS OR paramedics OR "ambulance calls" OR "911 calls" OR "first responders" counts OR "reaching out" OR surveillance OR enumerating OR track OR tracking "opioid users" OR "opiate users" OR "heroin users" OR "fentanyl users" OR "opioid addiction" OR "opiate addiction" OR "heroin addiction" OR "fentanyl addiction""

overdose OR death OR coroner OR fatality counts OR "reaching out" OR surveillance OR enumerating OR track OR tracking "rough sleeper" OR "Sleeping rough" OR homeless OR homelessness OR unhoused

overdose OR death OR coroner OR fatality counts OR "reaching out" OR surveillance OR enumerating OR track OR tracking "drug users" OR "people who use drugs" OR "Drug addicts" OR "people who inject drugs" OR "people who use injection drugs" OR "substance users"

overdose OR death OR coroner OR fatality counts OR "reaching out" OR surveillance OR enumerating OR track OR tracking "rooming house" OR shelters OR encampment OR "community housing" OR "public housing"

overdose OR death OR coroner OR fatality counts OR "reaching out" OR surveillance OR enumerating OR track OR tracking "opioid users" OR "opiate users" OR "heroin users" OR "fentanyl users" OR "opioid addiction" OR "opiate addiction" OR "heroin addiction" OR "fentanyl addiction"
